# Neurogranin as a Reliable Biomarker for Synaptic Dysfunction in Alzheimer’s Disease

**DOI:** 10.3390/diagnostics11122339

**Published:** 2021-12-12

**Authors:** Luisa Agnello, Bruna Lo Sasso, Matteo Vidali, Concetta Scazzone, Tommaso Piccoli, Caterina Maria Gambino, Giulia Bivona, Rosaria Vincenza Giglio, Anna Maria Ciaccio, Vincenzo La Bella, Marcello Ciaccio

**Affiliations:** 1 Department of Biomedicine, Neurosciences and Advanced Diagnostics, Institute of Clinical Biochemistry, Clinical Molecular Medicine and Clinical Laboratory Medicine, University of Palermo, 90127 Palermo, Italy; luisa.agnello@unipa.it (L.A.); bruna.losasso@unipa.it (B.L.S.); concetta.scazzone@unipa.it (C.S.); cmgambino@libero.it (C.M.G.); giulia.bivona@unipa.it (G.B.); giglio.rosaria.vincenza@gmail.com (R.V.G.); 2 Department of Laboratory Medicine, Azienda Ospedaliera Universitaria Policlinico “P. Giaccone”, 90127 Palermo, Italy; 3 Foundation IRCCS Ca’ Granda Ospedale Maggiore Policlinico, 20122 Milan, Italy; matteo.vidali@gmail.com; 4 Unit of Neurology, Department of Biomedicine, Neurosciences and Advanced Diagnostics, University of Palermo, 90127 Palermo, Italy; tommaso.piccoli@unipa.it; 5 Unit of Clinical Biochemistry, University of Palermo, 90127 Palermo, Italy; annamaria.ciaccio@unipa.it; 6 ALS Clinical Research Center, Department of Biomedicine, Neuroscience and Advanced Diagnostics, University of Palermo, 90129 Palermo, Italy; vincenzo.labella@unipa.it

**Keywords:** RC3, biomarkers, neurodegeneration, controls, diagnosis, prognosis, tau

## Abstract

(1) Background: Neurogranin is a post-synaptic protein expressed in the neurons of the hippocampus and cerebral cortex. It has been recently proposed as a promising biomarker of synaptic dysfunction, especially in Alzheimer’s disease (AD). However, more efforts are needed before introducing it in clinical practice, including the definition of its reference interval (RI). The aim of the study was to establish the RI of cerebrospinal fluid (CSF) neurogranin levels in controls and individuals with non-neurodegenerative neurological diseases; (2) We included a total of 136 individuals that were sub-grouped as follows: AD patients (*n* = 33), patients with non-neurodegenerative neurological diseases (*n* = 70) and controls (33). We measured CSF neurogranin levels by a commercial ELISA kit. CSF RI of neurogranin was calculated by a robust method; (3) Results: AD patients showed increased levels of neurogranin. We also found that neurogranin was significantly correlated with T-tau, P-tau and mini mental state examination in AD patients. The lower and upper reference limits of the RI were 2.9 (90%CI 0.1–10.8) and 679 (90%CI 595–779), respectively; (4) Conclusion: This is the first study establishing the RI of CSF neurogranin.

## 1. Introduction

Neurogranin is a calmodulin-binding protein discovered in 1990, but only in recent years it gained attention as a potential biomarker of neurodegeneration [1]. The term “neurogranin” refers to its expression in granule-like structures within excitatory neurons of the hippocampus and cerebral cortex [2]. It is a post-synaptic protein with a pivotal role in regulating synaptic plasticity and function [3,4]. Neurogranin knockout mice display a decrease in long-term potentiation (LTP) induction and cognition, while upregulation promotes LTP and improves cognitive performance [5,6].

In the last few decades, research has focused on the possible role of neurogranin as a biomarker for synaptic dysfunction in neurodegenerative diseases, such as Alzheimer’s disease (AD) [7,8]. Synaptic dysfunction, indeed, represents an important phenomenon in AD pathophysiology, which occurs early in the disease course, and leads to reduced cognitive function [9]. Neurogranin levels have been found to be markedly reduced in the frontal cortex and hippocampus, whereas they are found to be increased in the CSF of AD patients, indicating loss of post-synaptic elements in the extracellular space [10,11,12,13]. Although some authors failed to find an association between neurogranin and AD, most of the studies showed that it is a reliable biomarker of synaptic dysfunction, especially in AD, as summarized in two recently published meta-analyses [14,15]. Additionally, a correlation between CSF neurogranin levels and brain atrophy, as well as amyloid load and cognitive decline, has also been reported [16]. Finally, increased CSF neurogranin levels have been proved to be predictive of progression to AD in patients with mild cognitive impairment (MCI) [17]. Taken together, evidence in the literature supports the role of neurogranin as a biomarker of synaptic dysfunction in patients with AD [18]. Accordingly, CSF neurogranin has been proposed to be integrated as a potential new biomarker of synaptic degeneration and loss in the (N) group of the novel criteria for the diagnosis of AD, which was published by the National Institute on Aging and Alzheimer’s Association’s (NIA-AA) research framework [19]. However, there are still some limitations in transferring neurogranin from bench to bedside, including the lack of a reference interval (RI) and decisional cut-off value. Indeed, establishing a RI and decisional cut off of neurogranin could help to appropriately evaluate the clinical usefulness of such biomarker in patients with neurodegenerative diseases and promote its introduction in clinical practice.

The aim of this study was to evaluate the role of neurogranin as a biomarker of synaptic dysfunction, especially in AD patients, for its potential diagnostic application.

## 2. Materials and Methods

### 2.1. Study Population

We performed a retrospective observational study at the Palermo University Hospital “P. Giaccone” on a population consisting of controls and patients with NNND, attending the ALS Clinical Research Center, Palermo, from 2000 to 2020, who underwent lumbar puncture for CSF analysis as part of their diagnostic evaluation. We also included AD patients enrolled at the Unit of Neurology, A.U.O.P “P. Giaccone”, Palermo, Italy. The diagnosis of AD and NNND was achieved by an expert neurologist based on medical history, clinical examination, neuropsychological testing, neuroimaging, fluorodeoxyglucose PET and CSF biomarker findings, according to the clinical diagnostic criteria of McKhann et al. and Albert et al. [20,21].

All the clinical and biological assessments were carried out in accordance with the Declaration of Helsinki and its amendments. The study protocol was approved by the Ethics Committee of the University Hospital of Palermo, and all participants gave written, informed consent that contained the following statement: “the biological material may also be used for research purposes”.

### 2.2. CSF Analysis

The CSF samples were collected between 8:00 a.m. and 10:00 a.m. from fasted patients, and were labeled to ensure anonymity. Specifically, the CSF was obtained by a lumbar puncture at the L3/4 or L4/5 interspace using a 21-gauge needle. It was collected in polypropylene tubes, centrifuged at 500× *g* for 20 min, aliquoted in propylene tubes and stored at −80 °C until analysis, according to international consensus protocols [22].

CSF neurogranin levels were measured by a commercially available ELISA kit (Euroimmun, Lübeck, Germany), according to the manufacturer’s instructions.

The CSF β42, β40, P-tau and T-tau levels were measured by chemiluminescence enzyme immunoassay (CLEIA) (Lumipulse GpTau 181 and Lumipulse G Total Tau, Fujirebio Inc. Europe, Gent, Belgium) on a fully automated platform (Lumipulse G1200 analyzer, Fu-jirebio Inc. Europe, Gent, Belgium) according to the manufacturer’s instructions.

### 2.3. Statistical Analysis

Statistical analyses were performed by SPSS statistical software v.17.0 (SPSS Inc., Chicago, IL, USA), R Language v.4.0.3 (R Foundation for Statistical Computing, Vienna, Austria) and MedCalc Statistical Software v. 20.011 (MedCalc Software Ltd., Ostend, Belgium). Normality distribution was assessed preliminarily by Q–Q plot and the Shapiro–Wilk test. Quantitative variables were expressed by the median and interquartile range (IQR), while qualitative variables were expressed as absolute and relative frequencies. Differences between the groups, for continuous variables, were estimated by the nonparametric Kruskal–Wallis test (if >2 groups) or Mann–Whitney U-test with Holm–Bonferroni’s method for multiple comparisons. With the Holm–Bonferroni method, a comparison was considered statistically significant if the *p* value was lower than 0.05/(N − r + 1), where N was the number of *p* values (in this study N = 15) and r was the rank of the sorted *p* values, ordered lowest to highest. The association between quantitative variables was evaluated by the nonparametric Spearman’s rank-order correlation. Diagnostic accuracy for the diagnosis of AD was evaluated by ROC curve analysis and reported as AUC with 95% confidence intervals calculated by the DeLong method. The best cut off was evaluated by the Youden index. The association between Alzheimer diagnosis and neurogranin levels was also evaluated by multivariate logistic regression.

RI was calculated by the robust method, an iterative process based on robust measures of location and spread, following the Box–Cox transformation (lambda = 0.461, shift = 0). The 90% CI of the reference interval was calculated by the bootstrap percentile method (10,000 bootstrap replicates). Outlier detection was performed on raw data using Hampel’s test for outliers.

## 3. Results

One hundred and thirty-six subjects were enrolled in this study. They were subgrouped, according to previous clinical diagnosis, into AD (*n* = 33, 24.3%); patients with NNNDs, including patients with cerebrovascular diseases (*n* = 12, 8.8%), such as vascular encephalopathy and cerebral aneurysm, inflammatory CNS diseases (*n* = 10, 7.4%), such as myelitis and myeloradicoloneurites, peripheral neuropathy (*n* = 41, 30.1%), such as muscular dystrophy and multifocal motor neuropathy, other neurological diseases (*n* = 7, 5.1%), such as epilepsy and brain cancer; and controls (*n* = 33, 24.3%). Demographic characteristics (sex and age) are shown in Table 1.

**Figure 1:**
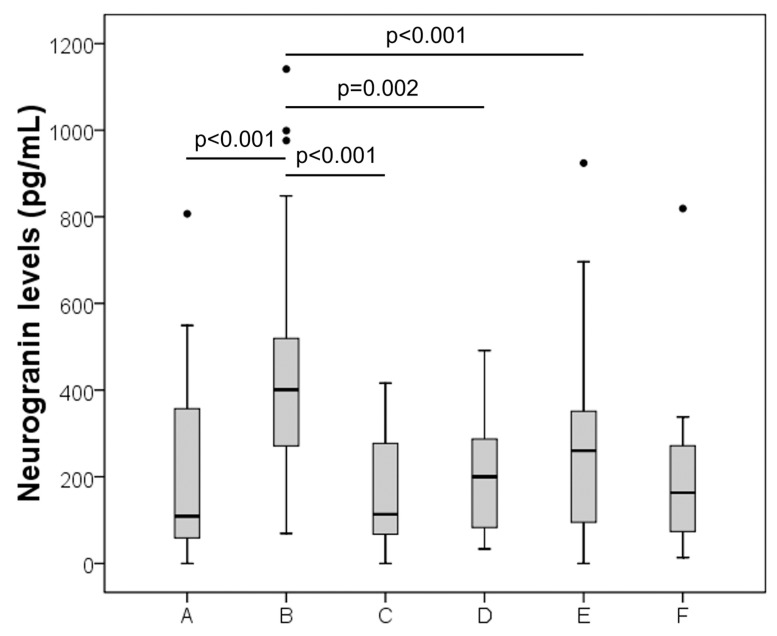
Boxplots of neurogranin levels in the groups investigated. A (controls), B (Alzheimer), C (cerebrovascular disease), D (inflammatory CNS disease), E (peripheral neuropathy) and F (other neurological diseases).

Neurogranin levels were significantly different among the groups (overall Kruskal–Wallis test *p* < 0.001) (Figure 1). In particular, taking into account Holm–Bonferroni’s method for multiple comparisons, AD patients displayed significantly higher median neurogranin levels than the controls (*p* < 0.001) and patients with cerebrovascular diseases (*p* < 0.001), inflammatory CNS diseases (*p* = 0.002) and peripheral neuropathy (*p* < 0.001). For the comparison of AD vs. other neurological diseases, the *p* value obtained (*p* = 0.015) was higher than the critical limit for significance calculated by the Holm–Bonferroni method (limit = 0.0045), which was then considered not significant. All other *p* values obtained were higher than 0.05 and ranged from 0.121 to 0.921. Neurogranin levels were also significantly higher in AD patients (*n* = 33) than patients of all NNNDs grouped together (*n* = 70) (median 401 vs. 212; *p* < 0.001). Moreover, a multivariate logistic regression using neurogranin, age and sex, confirmed that neurogranin was independently associated with AD (OR 1.004, 95%CI 1.001–1.006). Similar results were obtained excluding controls from the multivariate model (OR 1.003, 95%CI 1.001–1.006).

The RI of neurogranin was calculated in the population, including both controls and patients with NNNDs (*n* = 103). Three outliers were identified and eliminated (respectively, 807, 819 and 924). Using the remaining data (*n* = 100), the lower and upper reference limits of the RI were 2.9 (90%CI 0.1–10.8) and 679 (90%CI 595–779), respectively.

**Figure 2:**
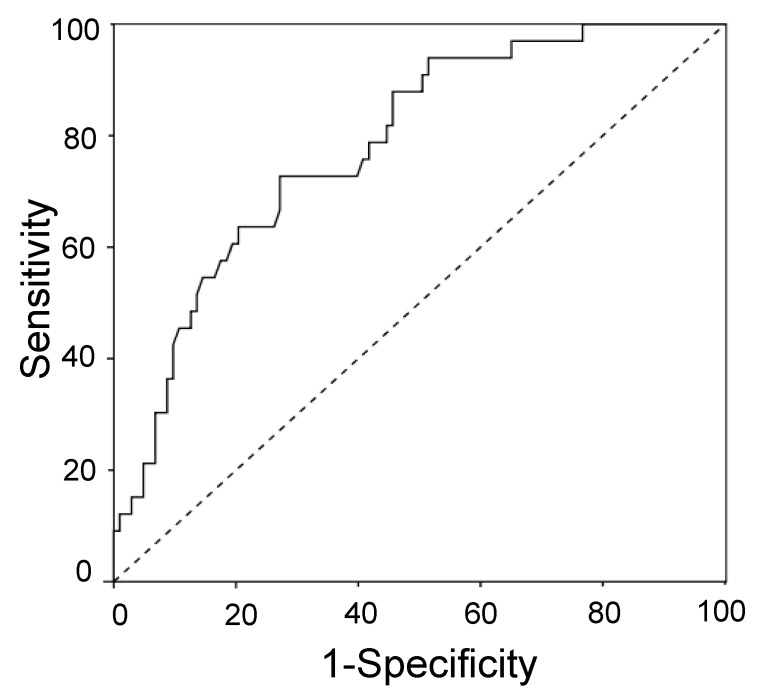
ROC curve of neurogranin for AD diagnosis. Dashed line indicates reference line.

The diagnostic performances of neurogranin for AD were evaluated by receiver operating characteristic (ROC) curve analysis (Figure 2). The area under the curve (AUC) was 0.78 (95%CI 0.69–0.87). According to Youden’s index, the best-calculated cut off was 319 pg/mL. At this cut off, sensitivity, specificity, positive predictive value and negative predictive values were 0.73, 0.73, 0.46 and 0.89, respectively.

Levels of CSF core biomarkers, namely amyloid β1-42 (β42), β1-40 (β40), total tau (T-tau), phosphorylated tau at threonine 181 (P-tau) and amyloid β1-42/ β1-40 ratio (β42/40 ratio), were further evaluated in AD patients. Neurogranin levels were found to be associated to varying extents with T-tau (rho = 0.808, *p* < 0.001), P-tau (rho = 0.655, *p* < 0.001) and β42/40 ratio (rho = −0.592, *p* = 0.001) (Table 2). Moreover, neurogranin was also found to be moderately and inversely associated with Mini-Mental State examination (MMSE) score (rho = −0.474; *p* = 0.012).

## 4. Discussion

Neurodegenerative diseases are an important health burden and their early identification is crucial, but this is still challenging today. CSF biomarkers represent precious tools for assessing neurodegenerative disorders, providing in vivo information on the underlying pathology [23,24,25,26,27]. Specifically, CSF is an ideal biofluid due to its proximity to the brain parenchyma, the lower intrinsic protease activity than blood, the moderately low cost in comparison to positron emission tomography (PET) imaging and the safety of lumbar puncture. To date, CSF biomarkers, including β42, β42/40 ratio, T-tau and P-tau, have been integrated in the diagnostic work-up of AD [19]. Neurogranin has recently emerged as a promising biomarker of synaptic dysfunction, especially in AD. Thus, it could provide integrative information to the core CSF biomarkers for AD management. It could also give important information for longitudinal monitoring of disease progression and drug effects on synaptic degeneration in clinical trials of disease-modifying therapies for AD.

It is noteworthy that although literature evidence encourages the introduction of neurogranin as biomarker of synaptic dysfunction, efforts are still required before implementing it in clinical practice. An essential issue is the definition of its RI. Indeed, the clinical laboratory test result has no value in isolation and, consequently, RI and decisional cut-off value are mandatory for appropriately interpreting the laboratory data [28,29,30,31,32]. However, especially for CSF biomarkers, establishing an RI is an arduous task due to the difficulties of obtaining such biological fluid in healthy individuals. In this study, we established the RI of neurogranin in a population of controls and individuals with non-neurodegenerative neurological diseases, which were not characterized by synaptic degeneration and, consequently, should not influence neurogranin levels. We applied the robust method, as suggested by the IFCC/CLSI document, when the number of individuals was lower than 120 [33]. The lower and upper reference limits of the RI, calculated by the robust method with bootstrapped 90% CI, were 2.9 (90%CI 0.25–10.1) and 679 (90%CI 595–779), respectively. In addition, we established 319 pg/mL as the decisional cut-off value of neurogranin for diagnosing AD. At this cut-off value, neurogranin showed good diagnostic accuracy, with an AUC of 0.78, and a sensitivity and specificity of 0.73. Finally, we found a strong correlation between neurogranin and CSF biomarkers of synapsis loss, T-tau and P-tau, as well as with MMSE. Whereas synapse loss and neuronal degeneration are pathogenic mechanisms interrelated in AD, it has been suggested that the evaluation of biomarkers reflecting both processes should be performed. Overall, our findings support the use of neurogranin as a biomarker of synaptic dysfunction.

In this study, we included only patients with AD, as a model of neurodegenerative diseases. Indeed, AD represents the most common form of dementia in the general population worldwide. Additionally, we did not compare AD with other dementia or neurodegenerative diseases, such as Lewy bodies dementia and Parkinson’s disease. Thus, we could not state that neurogranin is a specific biomarker of AD and further studies must be performed to assess whether neurogranin could be used as a biomarker for differential diagnosis among neurodegenerative diseases. Recently, Willemse et al. showed that neurogranin did not differentiate AD from non-AD dementia in two dementia cohorts, one included clinical AD and non-AD patients with high CSF tau levels and the other included patients with a definite post-mortem diagnosis, independently from CSF biomarkers [34]. The authors concluded that neurogranin could reflect a general pathophysiological process of synaptic degeneration and it was not specific for AD. On the contrary, Portelius et al. found that neurogranin was significantly increased in AD patients compared with patients with non-AD neurodegenerative diseases, including Parkinson’s disease and frontotemporal dementia [35]. Additionally, they showed that increased levels of neurogranin were associated with greater Aβ neuritic plaque and tau tangle pathology scores. Thus, the authors concluded that neurogranin could be a specific biomarker of AD. Overall, a definite conclusion on the specificity of neurogranin for AD could not be drawn, and further studies are required to address such question. To date, neurogranin certainly represents a biomarker of synaptic degeneration in AD.

Our findings are in accordance with previous studies, which showed good accuracy of neurogranin for AD, with an AUC ranging from 0.696 to 0.85 (Table 3). It is noteworthy that the decisional cut-off value, as well as the sensitivity and specificity, have not been specified in most of the studies, making it difficult to compare findings among studies.

The limitation and strengths in our study must be mentioned. The small number of AD is the main limitation, while the well-characterized patient groups, the use of a validated assay and the appropriate pre-analytical sample handling represent strengths in our study. It is noteworthy that the established cut-off value of neurogranin is based only on statistical calculation (Youden index). When considering the RI, a different cut off maybe selected based on the desired specificity and/or sensitivity. The validity of the cut-off value established in our study shall be confirmed in further studies by using an external validation cohort, including patients classified according to laboratory and clinical findings.

To the best of our knowledge, this is the first study defining the RIs of CSF neurogranin levels. We measured CSF neurogranin by a commercially available ELISA kit. Thus, it is plausible that using different ELISA kits or analytical methods, the RI could be slightly different. Thus, the RI established in our study can be transferred to other laboratories only after appropriate verification [28].

## Figures and Tables

**Table 1:** Demographic characteristics and neurogranin levels.

Group	Sex, M	Age (Median, IQR)	Neurogranin, pg/mL (Median, IQR)
AD (*n* = 33)	52%	71 (66–77)	401 (270–521)
Cerebrovascular diseases (*n* = 12)	75%	60 (52–67)	114 (58–281)
Inflammatory CNS diseases (*n* = 10)	30%	64 (58–76)	200 (76–303)
Peripheral Neuropathy (*n* = 41)	61%	60 (48–71)	260 (89–352)
Other neurological diseases (*n* = 7)	43%	63 (48–72)	163 (38–338)
Controls (*n* = 33)	58%	52 (39–67)	109 (54–373)

AD, Alzheimer’s disease; CNS, central nervous system; M, male sex; IQR, inter quartile range.

**Table 2:** Correlation analysis among CSF biomarkers in AD patients.

	Neurogranin	T-tau	P-tau	β42	β40	β42/40 Ratio
**Neurogranin**		0.808 *p* < 0.001	0.655 *p* < 0.001	0.053 *p* = 0.780	0.321 *p* = 0.118	−0.592 *p* = 0.001
**T-tau**			0.810 *p* < 0.001	−0.030 *p* = 0.877	0.254 *p* = 0.221	−0.492 *p* = 0.006
**P-tau**				0.013 *p* = 0.947	0.333 *p* = 0.104	−0.563 *p* = 0.001
**β42**					0.764 *p* < 0.001	0.463 *p* = 0.010
**β40**						−0.024 *p* = 0.908
**β42/40 ratio**						

**Table 3:** Studies on the accuracy of CSF neurogranin for AD diagnosis.

	Study Population	Assay	AUC	Sensitivity	Specificity	Optimal Cut-Off Value (pg/mL)
Xue et al. [36]	111 cognitively-normal controls, 193 MCI, 95 AD	Electrochemiluminescence technology using Ng7	0.71	NA	NA	NA
Fan et al. [37]	65 cognitively-normal controls, 65 AD	Electrochemiluminescence technology using Ng7	0.783	NA	NA	NA
Galasko et al. [38]	90 normal controls, 46 AD	ELISA (EUROIMMUN, Lübeck, Germany)	0.504	0.200 (0.022–0.311)	0.854 (0.640–0.932)	167.78
Schipke et al. [39]	20 MDD, 20 AD	In-house ELISA	0.696	NA	NA	NA
Mattsson et al. [40]	93 controls, 93 AD	In-house ELISA	0.85	NA	NA	254.7
Tarawneh et al. [41]	207 controls, 95 AD	2-site immunoassay uses an affinity-efficient trapping and purification technique for polyclonal antibodies	0.71	NA	NA	NA
Janelidze et al. [42]	74 AD	In-house ELISA	0.761	NA	NA	NA

MCI, mild cognitive impairment; AD, Alzheimer’s disease; NA, not available; ELISA, enzyme-linked immunosorbent assay; MDD, major depressive disorder.

## Data Availability

The datasets generated during and/or analyzed during the current study are available from the corresponding author upon reasonable request.
